# Adaptive Damping Log-Domain Message-Passing Algorithm for FTN-OTFS in V2X Communications

**DOI:** 10.3390/s25123692

**Published:** 2025-06-12

**Authors:** Hui Xu, Chaorong Zhang, Qingying Wu, Benjamin K. Ng, Chan-Tong Lam

**Affiliations:** Faculty of Applied Sciences, Macao Polytechnic University, Macao 999078, China; hui.xu@mpu.edu.mo (H.X.); p2314785@mpu.edu.mo (C.Z.); p2009037@mpu.edu.mo (Q.W.); ctlam@mpu.edu.mo (C.-T.L.)

**Keywords:** orthogonal time frequency space (OTFS), vehicle-to-everything (V2X), faster-than-Nyquist (FTN) signaling, spectrally efficient frequency division multiplexing (SEFDM)

## Abstract

To enable highly reliable and spectrum-efficient vehicle-to-everything (V2X) communications under conditions with severe Doppler effects and rapidly time-varying channels, we propose a novel faster-than-Nyquist orthogonal time frequency space (FTN-OTFS) modulation scheme. In this scheme, FTN signaling is integrated with spectrally efficient frequency division multiplexing (SEFDM) within the OTFS framework, enabling a higher symbol-transmission density within a fixed time–frequency resource block and thus enhancing spectral efficiency without increasing the occupied bandwidth. An analytical input–output model is derived in both the delay–Doppler and time–frequency domains. To further enhance numerical stability, an improved detection algorithm called adaptive damping log-domain message-passing (ADL-MP) is developed for the proposed scheme. Simulation results demonstrate that the proposed scheme achieves robust and reliable performance in high-mobility scenarios and that the proposed algorithm consistently outperforms conventional methods in terms of bit error rate (BER) under both the extended vehicular A (EVA) model and the high-speed train (HST) scenario, confirming its effectiveness and superiority for V2X communications.

## 1. Introduction

With the rapid advancement of intelligent transportation systems [1] and vehicle-to-everything (V2X) communication [2,3,4], wireless communication systems in high-mobility environments face increasingly stringent demands for high reliability, low latency, and high spectral efficiency [5]. In typical V2X scenarios, vehicles often travel at high speeds and navigate complex urban landscapes, resulting in rapidly time-varying wireless channels with significant Doppler shifts [6]. These characteristics present substantial challenges for designing physical-layer transmission schemes.

Orthogonal frequency division multiplexing (OFDM) is well known for its excellent performance in time-invariant channels and is widely adopted in modern wireless systems due to its high spectral efficiency [7]. However, the performance of OFDM significantly degrades in contexts with rapidly time-varying channels, such as those encountered in high-mobility V2X communications [8]. In high-mobility scenarios, rapid Doppler shifts disrupt subcarrier orthogonality, leading to severe intercarrier interference (ICI) and intersymbol interference (ISI) [9]. These impairments degrade OFDM system throughput and reliability, posing significant challenges in meeting the requirements of V2X applications for low latency and high reliability. To overcome these limitations, [10] introduced orthogonal time frequency space (OTFS), a two-dimensional modulation technique that maps information symbols onto the delay-Doppler (DD) domain. In [11], the discrete-time model of OTFS was derived, demonstrating that OTFS can be implemented by incorporating appropriate preprocessing and postprocessing operations into the conventional OFDM framework. By exploiting the reduced channel variability in the DD domain, OTFS can harness both time and frequency diversity more effectively than can OFDM, resulting in enhanced transmission performance. Furthermore, in the DD domain, time-varying channels are transformed into quasi-static and sparse representations, making OTFS highly suitable for high-mobility applications. However, mapping symbols to the DD domain inevitably introduces inter-Doppler interference (IDI), which poses new challenges for receiver design. To achieve full time-frequency diversity and robustly suppress interference from various sources, efficient detection algorithms are essential. The symbol-wise maximum a posteriori (MAP) algorithm achieves optimal performance in interference channels [12], but its computational complexity grows exponentially with the OTFS frame size, making it impractical for real-world systems. To address this issue, a message-passing detection algorithm based on the factor graph framework was proposed in [13]; in that algorithm, inter-symbol interference is modeled using Gaussian approximations. It has been shown that the message-passing algorithm can achieve near-optimal performance with significantly reduced complexity. Furthermore, by employing appropriate phase shifts, the message-passing algorithm can effectively eliminate ICI and ISI, and through focus on the most significant interference terms, the impact of IDI can be mitigated [14,15]. Consequently, the message-passing algorithm can effectively compensate for a wide range of channel Doppler spreads [16]. Despite these advances in detection algorithms, the data rate of conventional OTFS modulation remains substantially limited by its reliance on ideal bi-orthogonal pulses.

Faster-than-Nyquist (FTN) signaling is a prominent non-orthogonal transmission technique that deliberately violates the Nyquist criterion by compressing the symbol interval during pulse shaping [17]. This approach enables the transmission of more symbols within a fixed bandwidth and improves the information rate but introduces ISI as a trade-off [18]. In [19], FTN signaling was extended to the frequency domain by applying it to OFDM systems in a method referred to as spectrally efficient frequency division multiplexing (SEFDM). By reducing the subcarrier spacing in OFDM, SEFDM enables the transmission of more information symbols within the same spectral resources. Fundamentally, SEFDM can be considered a multi-carrier implementation of FTN signaling.

To address the limitations of conventional OTFS and ensure reliable communication in high-mobility environments, the integration of FTN signaling with OTFS has emerged as a promising approach. This combination offers the potential to enhance the information rate and meet the demands of next-generation V2X communication systems, making it a key focus of research. In [20], an eigenvalue decomposition (EVD) precoded OTFS-based FTN architecture was proposed. In this approach, power-allocation coefficients were optimized for each frame to maximize mutual information under a total transmit power constraint. The study confirmed that the OTFS-based FTN scheme achieved higher spectral efficiency compared to the traditional OTFS scheme. In [21], a novel waveform design combining OTFS and FTN signaling was presented, employing a minimum mean square error (MMSE) equalizer to mitigate ISI introduced by FTN signaling. Furthermore, ref. [22] proposed a new FTN-OTFS system in which non-orthogonal pulses were jointly applied in the time-frequency (TF) domain. However, these studies primarily focused on system design and failed to address the development or application of effective nonlinear detection algorithms, which remained an open area for further investigation.

Against this background, this paper proposes a novel FTN-OTFS modulation scheme, derives the corresponding input–output models, and designs a new detection algorithm. To better reflect practical V2X scenarios, we consider both the extended vehicular A (EVA) and high-speed train (HST) channel models, further demonstrating the application potential of the proposed solution in real-world V2X environments. Simulation results show that the proposed detection algorithm achieves superior bit error rate (BER) performance and enhanced transmission reliability in highly dynamic V2X environments, highlighting its significant potential as a physical-layer solution for future high-mobility communications.

Specifically, the main contributions of our works can be summarized as follows:

- We propose a novel FTN-OTFS modulation scheme that combines SEFDM to compress subcarrier spacing in the frequency domain and root-raised-cosine pulse-shaped FTN signaling to break the Nyquist criterion in the time domain, thereby accelerating symbol transmission. By utilizing non-orthogonal techniques, the scheme significantly boosts system throughput under conditions with severe Doppler effects and fast time-varying channels in V2X scenarios.
- We derive analytically tractable input–output models for the proposed FTN-OTFS system in both the DD and TF domains, providing a solid theoretical foundation for the design of efficient receiver algorithms.
- We propose a novel adaptive damping log-domain message-passing (ADL-MP) detection algorithm, which incorporates a convergence-rate adaptive damping mechanism to improve both convergence speed and numerical stability. The complexity and convergence properties of the proposed algorithm are also analyzed.

The rest of this paper is organized as follows. Section 2 gives the system model, including the transmitter, channel model, and receiver. Section 3 details the proposed ADL-MP detection algorithm. Section 4 provides extensive simulation results. Section 5 comprises the conclusion.

## 2. System Model

**Figure 1:**
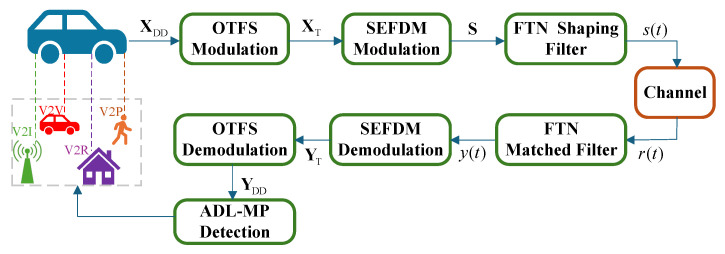
Block diagram of FTN-OTFS systems for V2X communications.

This section considers an FTN-OTFS transmission scheme with *N* symbols and *M* subcarriers that was designed for high-mobility V2X communication scenarios, such as those commonly encountered on highways. In this system, the subcarrier spacing is denoted by Δf and the symbol duration is given by T=1/Δf. Consequently, each OTFS frame spans a total duration of NT and occupies a bandwidth of MΔf. The corresponding system model (As shown in Figure 1, V2X communication links are manifested in multiple modes, including but not limited to vehicle-to-infrastructure (V2I), vehicle-to-vehicle (V2V), vehicle-to-pedestrian (V2P), and vehicle-to-roadside unit (V2R). These links are typically characterized by highly time-varying channels, significant Doppler shifts, and pronounced delay spreads) is illustrated in Figure 1.

### 2.1. Transmitter

The information symbols in the DD domain are represented as $X_{\mathrm{DD}}\in C^{M\times N}$. Transmitter processing occurs in three main stages, namely OTFS modulation, SEFDM modulation, and FTN pulse shaping, which occur in sequence.

First, the time-domain signals are generated through OTFS modulation. To convert $X_{\mathrm{DD}}$ into the TF domain, the inverse symplectic finite Fourier transform (ISFFT) is applied. Specifically, the ISFFT performs an *M*-point fast Fourier transform (FFT) along the delay dimension and an *N*-point IFFT along the Doppler dimension, resulting in the TF-domain transmit symbols. Subsequently, an *M*-point inverse fast Fourier transform (IFFT) is applied to the TF-domain symbols to obtain the time-domain signal, which can be expressed as follows:

(1) $$X_{T}=F_{M}^{H}\left(F_{M}X_{\mathrm{DD}}F_{N}^{H}\right),$$

where $F_{M}$ represents the *M*-point discrete Fourier transform (DFT) matrix, while $F_{N}^{H}$ and $F_{M}^{H}$ denote the *N*-point and *M*-point inverse discrete Fourier transform (IDFT) matrices, respectively.

Next, the time-domain signal $X_{T}$ undergoes modulation using SEFDM. In SEFDM, the subcarrier spacing is compressed to αΔf, where α represents the SEFDM compression factor, satisfying 0<α<1. The transmitted signal in SEFDM can be expressed as [23]

(2) $$S=F_{\alpha }^{-1}X_{T}=F_{\alpha }^{-1}X_{\mathrm{DD}}F_{N}^{H},$$

where $F_{\alpha }^{-1}$ represents the SEFDM modulation matrix, which is the inverse fractional Fourier transform (IFrFT) matrix and can be expressed as

(3) $$F_{\alpha }^{-1}=\left[\begin{array}{cccc} 1 & 1 & \ldots & 1\\ 1 & \varepsilon^{\alpha } & \ldots & \varepsilon^{\alpha (M-1)}\\ \vdots & \vdots & \ddots & \vdots \\ 1 & \varepsilon^{\alpha (M-1)} & \ldots & \varepsilon^{\alpha (M-1)(M-1)} \end{array}\right],$$

where $\varepsilon =e^{j2\pi /M}$. It is worth noting that when α=1, the IFrFT becomes equivalent to the conventional IFFT. Then, S can be vectorized as

(4) $$s=\mathrm{vec}\left(S\right)=\left(F_{N}^{H}⊗F_{\alpha }^{-1}\right)x_{\mathrm{DD}},$$

where ⊗ is the Kronecker product.

Subsequently, the transmitted signal s is then passed through the FTN pulse-shaping filter p(t). We assume that the pulse-shaping filter has unit energy; i.e., $\int_{-\infty }^{+\infty }{\left|p\left(t\right)\right|}^{2}dt=1$. The FTN-OTFS transmitted signal is given by

(5) $$s\left(t\right)=\sum_{n=0}^{MN-1}s_{n}p\left(t-n\beta T_{0}\right),$$

where $\beta T_{0}=T/M$, β is the FTN compression factor, and $T_{0}$ is the symbol interval defined by the ISI-free Nyquist criterion.

### 2.2. Channel Model

Consider a time-varying channel characterized by *P* propagation paths. For the *p*th path, $h_{p}$, $\tau_{p}$, and $\nu_{p}$ represent the channel gain, delay, and Doppler shift, respectively. The DD domain-channel response can be written as  

(6) $$h\left(\tau ,\nu \right)=\sum_{p=1}^{P}h_{p}\delta \left(\tau -\tau_{p}\right)\delta \left(\nu -\nu_{p}\right),$$

where δ(·) is Dirac’s delta function. The delay and Doppler shift are given by

(7) $$\tau_{p}=\frac{l_{p}}{M\Delta f},\;\nu_{p}=\frac{k_{p}+\kappa_{p}}{NT},$$

where the delay indices $l_{p}$ and Doppler shift indices $k_{p}$ are integers and $\kappa_{p}\in \left(-1/2,1/2\right]$ represents the fractional part of the Doppler shift.

### 2.3. Receiver

Following passage through the time-varying channel, the received signal r(t) is given by

(8) $$\begin{array}{cc} r(t) & =\int_{-\infty }^{\infty }\int_{-\infty }^{\infty }h\left(\tau ,\nu \right)e^{j2\pi \nu (t-\tau )}s\left(t-\tau \right)d\tau \;d\nu +n\left(t\right)\\ \; & =\sum_{p=1}^{P}h_{p}s\left(t-\tau_{p}\right)e^{j2\pi \nu_{p}\left(t-\tau_{p}\right)}+n\left(t\right), \end{array}$$

where n(t) is the the complex-valued additive white Gaussian noise (AWGN) with a mean of zero and variance $N_{0}$.

The received signal r(t) is then passed through an FTN matched filter $p^{*}\left(-t\right)$. The matched-filtered output can be expressed as

(9) $$\begin{array}{cc} y(t)= & r\left(t\right)★p^{*}\left(-t\right)\\ = & \left[\sum_{p=0}^{P-1}h_{p}e^{j2\pi \frac{\left(k_{p}+\kappa_{p}\right)\left(t-l_{p}\beta T_{0}\right)}{MN\beta T_{0}}}s\left(t-l_{p}\beta T_{0}\right)+n\left(t\right)\right]★\;p^{*}\left(-t\right)\\ = & \sum_{p=0}^{P-1}\sum_{n=0}^{MN-1}h_{p}e^{j2\pi \frac{\left(k_{p}+\kappa_{p}\right)\left(t-l_{p}\beta T_{0}\right)}{MN\beta T_{0}}}s_{n}g\left(t-\left(n+l_{p}\right)\beta T_{0}\right)+\eta \left(t\right), \end{array}$$

where ★ denotes the convolution operation. Specifically, we define $g\left(t\right)≜p\left(t\right)★p^{*}\left(-t\right)$ and $\eta \left(t\right)≜n\left(t\right)★p^{*}\left(-t\right)$ [24]. Then, y(t) is sampled with interval $\beta T_{0}$ and the discrete received signal is obtained as

(10) $$y_{T}=Hs+\eta ,$$

From (9), the entry in the *k*th-row and *m*th-column of effective channel matrix $H\in C^{MN\times MN}$ can be written as

(11) $$H\left(k,m\right)=\sum_{p=0}^{P-1}h_{p}e^{j2\pi \frac{\left(k_{p}+\kappa_{p}\right)\left(k-l_{p}\right)}{MN}}g\left(\left(k-m-l_{p}\right)\beta T_{0}\right).$$

Next, $y_{T}$ is rearranged into an M×N matrix and SEFDM demodulation is performed. The demodulated received signal is given by

(12) $$Y_{T}=F_{\alpha }{\mathrm{vec}}^{-1}\left(y_{T}\right).$$

Then, OTFS demodulation is applied to $Y_{T}$ to obtain the DD domain received signal $Y_{\mathrm{DD}}$. Specifically, an *M*-point FFT operation is first applied to each column of $Y_{T}$. Subsequently, the SFFT is performed; this involves an *M*-point IFFT applied to the columns, followed by an *N*-point FFT applied to the rows. Therefore, YDD can be expressed as

(13) $$Y_{\mathrm{DD}}=F_{M}^{H}F_{M}Y_{T}F_{N}=F_{\alpha }{\mathrm{vec}}^{-1}\left(y_{T}\right)F_{N}.$$

where $Y_{\mathrm{DD}}\in C^{M\times N}$, and whose vectorized form can be expressed as

(14) $$y_{\mathrm{DD}}=\mathrm{vec}\left(Y_{\mathrm{DD}}\right)=\left(F_{N}⊗F_{\alpha }\right)y_{T}.$$

Finally, the relationship between the transmitted vector $x_{\mathrm{DD}}$ and the received vector $y_{\mathrm{DD}}$ is given by

(15) $$\begin{array}{cc} y_{\mathrm{DD}} & =\left(F_{N}⊗F_{\alpha }\right)H\left(F_{N}^{H}⊗F_{\alpha }^{-1}\right)x_{\mathrm{DD}}+\left(F_{N}⊗F_{\alpha }\right)\eta \\ \; & =H_{\mathrm{DD}}x_{\mathrm{DD}}+\eta_{\mathrm{DD}}. \end{array}$$

where $x_{\mathrm{DD}}$, $y_{\mathrm{DD}}$, and $\eta_{\mathrm{DD}}$ are vectors of dimension NM×1. The DD domain channel matrix is defined as $H_{\mathrm{DD}}=\left(F_{N}⊗F_{\alpha }\right)H\left(F_{N}^{H}⊗F_{\alpha }^{-1}\right)$, and $H_{\mathrm{DD}}\in C^{MN\times MN}$.

## 3. Adaptive Damping Log-Domain Message-Passing Algorithm

The message-passing algorithm exploits the highly sparse and locally coupled structure of the time-varying channel. Instead of relying on cubic-complexity matrix inversion, it performs local probability updates with computational complexity that scales linearly with the frame size, yet still achieves performance close to that of the MAP detector. Specifically, the sparse and locally coupled structure of the time-varying channel is naturally represented as a factor graph. In this framework, the global inference problem is decomposed into a series of local, iterative probability updates between nodes. The message-passing algorithm models the detection process as message exchanges on a factor graph, with variable nodes representing transmitted symbols and observation nodes representing received signals. Due to channel sparsity, each observation node is connected to only a limited number of variable nodes, enabling efficient computation. By iteratively updating and exchanging probability messages between nodes, the algorithm approximates the marginal posterior probabilities of the transmitted symbols. This approach transforms a high-dimensional joint-detection problem into a set of manageable subproblems, achieving efficient and near-optimal detection performance with significantly reduced computational complexity. These characteristics of low complexity and near-optimality make message passing a natural foundation for V2X receivers, which must meet strict requirements for latency and reliability.

In this context, we propose an adaptive damping log-domain message passing (ADL-MP) detection algorithm, which leverages probabilistic message passing with log-sum-exp (LSE) normalization to effectively prevent exponential overflow and ensure numerical stability. By operating in the log-domain, the algorithm avoids the computational challenges associated with handling extremely large or small values, making it more reliable in practical implementations. Additionally, an adaptive damping factor is introduced to further optimize the performance of the algorithm. The damping factor is designed to accelerate the convergence of the iterative process while simultaneously enhancing the robustness of the algorithm, particularly under challenging or noisy conditions. The combination of log-domain normalization and adaptive damping provides a powerful framework for achieving both stability and efficiency in message-passing computations.

### 3.1. An Overview of the Message-Passing Algorithm

The input–output relationship in the system is expressed as in (15). The elements of the received signal vector $y_{\mathrm{DD}}$ are denoted by {y[d]∣1≤d≤NM}. Similarly, the elements of the channel matrix $H_{\mathrm{DD}}$ are denoted by {H[d,c]∣1≤d,c≤NM}, where H[d,c] corresponds to the channel response between the *c*th transmitted DD domain symbol and the *d*th received DD domain symbol. The transmitted signal vector $x_{\mathrm{DD}}$ is represented by its elements as {x[c]∣1≤c≤NM}. Furthermore, the noise vector $\eta_{\mathrm{DD}}$ is denoted by its elements as {η[d]∣1≤d≤NM}.

The channel matrix $H_{\mathrm{DD}}$ is sparse, reflecting the fact that only a limited number of propagation paths contribute to the received signal at each DD domain index. Specifically, I(d) and J(c) are defined as the sets of indices corresponding to the non-zero entries in the *d*th row and *c*th column of $H_{\mathrm{DD}}$, respectively. Both sets have a cardinality of *P*, indicating that there are *P* significant propagation paths for each transmitted or received symbol. This sparsity significantly reduces the computational complexity required for processing, as only a subset of the matrix elements needs to be considered.

Given this sparse structure, the system model described in (15) can be interpreted as a factor graph. The factor graph consists of NM observation nodes, corresponding to the elements of the received signal vector $y_{\mathrm{DD}}$, and NM variable nodes, corresponding to the elements of the transmitted signal vector $x_{\mathrm{DD}}$. In this graph, each observation node y[d] is directly connected to the variable nodes {x[c]∣c∈I(d)}, which represent the transmitted symbols contributing to the received signal at index *d*. Similarly, each variable node x[c] is connected to the observation nodes {y[d]∣d∈J(c)}, which represent the received signals that are influenced by the transmitted symbol at the index *c*. This factor graph representation provides a structured and intuitive way to model the input–output relationship of the system, capturing both the sparsity of the channel and the interactions between transmitted and received symbols. It is particularly useful for designing and analyzing message-passing algorithms, as it directly maps the system model onto a graphical framework that facilitates iterative processing and efficient computation.

From (15), the rule for joint MAP detection used to estimate the transmitted signals is formulated as

(16) $$\hat{x}=\underset{x_{\mathrm{DD}}\in A^{NM\times 1}}{\arg\max}\mathrm{Pr}\left(x_{\mathrm{DD}}|y_{\mathrm{DD}},H_{\mathrm{DD}}\right).$$

Since the computational complexity of MAP detection increases exponentially with NM, performing full MAP detection for large-scale systems becomes impractical. To address this, we consider performing MAP detection on a symbol-by-symbol basis, an approach that significantly reduces complexity while maintaining reasonable detection performance. The estimation symbol $\hat{x}\left[c\right]$ can be expressed as follows:  

(17) $$\begin{array}{cc} \hat{x}\left[c\right] & =\underset{a_{j}\in A}{\arg\max}\mathrm{Pr}\left(x\left[c\right]=a_{j}|y_{\mathrm{DD}},H_{\mathrm{DD}}\right)\\ \; & =\underset{a_{j}\in A}{\arg\max}\frac{1}{Q}\mathrm{Pr}\left(y_{\mathrm{DD}}|x\left[c\right]=a_{j},H_{\mathrm{DD}}\right)\\ \; & \approx \underset{a_{j}\in A}{\arg\max}\prod \mathrm{Pr}\left(y\left[d\right]|x\left[c\right]=a_{j},H_{\mathrm{DD}}\right). \end{array}$$

The MAP detection in (17) can be effectively realized using the message-passing algorithm. This algorithm operates iteratively, leveraging the structure of the factor graph to exchange information between observation nodes and variable nodes. In the message-passing algorithm, each observation node y[d] transmits to the connected variable nodes {x[c]∣c∈I(d)} a message consisting of the mean and variance of the Gaussian interference term. These messages encapsulate statistical information about the received signal and interference; this information is crucial for refining the estimates of the transmitted symbols. Conversely, each variable node x[c] exchanges information with its connected observation nodes y[d]∣d∈J(c). The message transmitted from a variable node takes the form of a probability mass function (PMF) denoted as $P_{c,d}=\left\{p_{c,d}\left(a_{j}\right)|a_{j}\in A\right\}$. Here, $P_{c,d}$ represents the likelihood of the transmitted symbol x[c] being equal to each candidate symbol $a_{j}$ in the constellation set A. This probabilistic representation enables the algorithm to incorporate uncertainty and refine the detection process iteratively.

**Figure 2:**
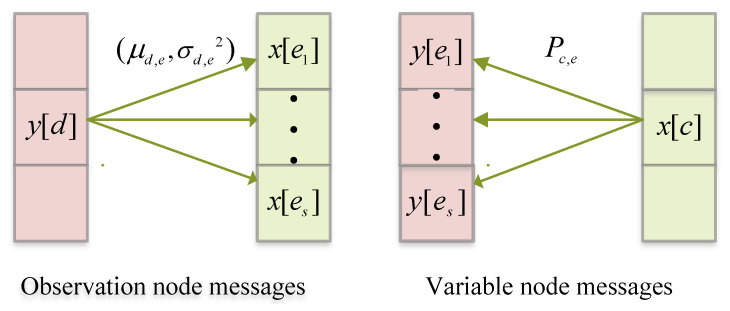
Messages in factor graph.

The entire message-passing process, which involves the exchange of messages between variable nodes and observation nodes, is visually represented in Figure 2. In this figure, the connections between nodes are shown as edges, illustrating the flow of information. For each observation node y[d], the set of edges linking it to variable nodes is denoted by $e_{1},e_{2},\ldots ,e_{S}=I\left(d\right)$, where *S* represents the number of connected variable nodes. Similarly, for each variable node x[c], the edges connecting it to observation nodes are represented by $e_{1},e_{2},\ldots ,e_{S}=J\left(c\right)$. This iterative exchange of messages between nodes ensures that the algorithm systematically updates its estimates of the transmitted symbols. The use of the MP algorithm is particularly advantageous in sparse systems in which the factor graph has a limited number of connections per node. This sparsity reduces the computational complexity, making the algorithm both efficient and scalable for large-scale systems. Moreover, by exploiting the statistical properties of the messages, the MP algorithm achieves robust performance, even in challenging scenarios with noise and interference.

The message-passing algorithm obtains the final detected signal through iterative computation. Assume that in the *i*th iteration, the messages passed from observation nodes to variable nodes are the mean $\mu_{d,c}^{i}$ and variance ${\left(\sigma_{d,c}^{i}\right)}^{2}$ of the interference term $\zeta_{d,c}^{i}$. This interference term is approximated as a Gaussian random variable, as described by [13]

(18) $$y\left[d\right]=x\left[c\right]H\left[d,c\right]+\underset{\zeta_{d,c}^{\left(i\right)}}{\underbrace{\sum_{e\in I(d),e\neq c}x\left[e\right]H\left[d,e\right]+z\left[d\right]}},$$

The mean $\mu_{d,c}^{i}$ and variance ${\left(\sigma_{d,c}^{i}\right)}^{2}$ of $\zeta_{d,c}^{i}$ are calculated as  

(19) $$\begin{array}{cc} \mu_{d,c}^{\left(i\right)}= & \sum_{e\in I(d),e\neq c}\sum_{j=1}^{Q}p_{e,d}^{\left(i-1\right)}\left(a_{j}\right)a_{j}H\left[d,e\right],\\ {\left(\sigma_{d,c}^{\left(i\right)}\right)}^{2}= & \sum_{e\in I(d),e\neq c}\left(\sum_{j=1}^{Q}p_{e,d}^{\left(i-1\right)}\left(a_{j}\right){\left|a_{j}\right|}^{2}{\left|H\left[d,e\right]\right|}^{2}\right.\\ \; & \left.-{\left|\sum_{j=1}^{Q}p_{e,d}^{\left(i-1\right)}\left(a_{j}\right)a_{j}H\left[d,e\right]\right|}^{2}\right)+\sigma^{2}. \end{array}$$

### 3.2. Message Updating from the Variable Node to the Observation Node via LSE Normalization and Adaptive Damping

The ADL-MP algorithm is detailed in Algorithm 1, wherein a step-by-step procedure for its implementation is presented. This algorithm operates iteratively over variable nodes and observation nodes within the factor graph structure to efficiently estimate transmitted symbols. For each variable node x[c] and each observation node y[e], the log-likelihood associated with the constellation point $a_{k}$ is defined as

(20) $$\ln\xi^{\left(i\right)}\left(e,c,k\right)=\frac{-{\left|y\left[e\right]-\mu_{e,c}^{\left(i\right)}-H_{e,c}a_{k}\right|}^{2}}{{\left(\sigma_{e,c}^{\left(i\right)}\right)}^{2}}.$$

The normalized probability transmitted from the variable node x[c] to the observation node y[d] is determined using the LSE normalization technique, which ensures numerical stability and avoids computational issues related to underflow or overflow. The expression for the normalized log-probability is given as

(21) $$\begin{array}{c} \ln{\tilde{p}}_{c,d}^{\left(i\right)}\left(a_{j}\right)=\sum_{e\in J(c),e\neq d}\left[\ln\xi^{\left(i\right)}\left(e,c,j\right)-\ln\left(\sum_{k=1}^{Q}\exp\left(\ln\xi^{\left(i\right)}\left(e,c,k\right)\right)\right)\right]. \end{array}$$

The message passed from the variable node x[c] to its connected observation node y[d] is represented by a PMF vector, which is denoted as $p_{c,d}^{\left(i\right)}$, The update process for $p_{c,d}^{\left(i\right)}$ is defined as

(22) $$\begin{array}{c} p_{c,d}^{\left(i\right)}\left(a_{j}\right)=\Delta^{\left(i\right)}\cdot \frac{\exp\left(\ln{\tilde{p}}_{c,d}^{\left(i\right)}\left(a_{j}\right)\right)}{\sum_{k=1}^{Q}\exp\left(\ln{\tilde{p}}_{c,d}^{\left(i\right)}\left(a_{k}\right)\right)}+\left(1-\Delta^{\left(i\right)}\right)\cdot p_{c,d}^{\left(i-1\right)}\left(a_{j}\right), \end{array}$$

(23) $$\Delta^{\left(i\right)}=\max\left(0,\min\left(1,\eta^{\left(i-1\right)}\right)\right),$$

where $\Delta^{\left(i\right)}$ represents the adaptive damping factor, while $\eta^{\left(i\right)}$ serves as the convergence indicator. The value of $\eta^{\left(i\right)}$ can be computed as

(24) $$\eta^{\left(i\right)}=\frac{1}{NM}\sum_{c=1}^{NM}I\left(\max_{a_{j}\in A}p_{c}^{\left(i\right)}\left(a_{j}\right)\geq 1-\gamma \right),$$

where I(·) is the indicator function, returning 1 when the condition holds and 0 otherwise; γ is a tolerance threshold for determining convergence confidence; and $p_{c}^{\left(i\right)}\left(a_{j}\right)$ denotes the posterior probability of symbol $a_{j}$ at variable node x[c] in the *i* th iteration, which can be calculated as

(25) $$p_{c}^{\left(i\right)}\left(a_{j}\right)=\prod_{d\in J(c)}\frac{\exp\left(\ln\xi^{\left(i\right)}\left(d,c,j\right)\right)}{\sum_{k=1}^{Q}\exp\left(\ln\xi^{\left(i\right)}\left(d,c,k\right)\right)}.$$

If $\eta^{\left(i\right)}>\eta^{\left(i-1\right)}$, the decision for the transmitted symbol is updated as follows:

(26) $$\hat{x}\left[c\right]=\underset{a_{j}\in A}{\arg\max}\;p_{c}^{\left(i\right)}\left(a_{j}\right),\;c=1,\ldots ,NM.$$

In the proposed algorithm, the decisions for the transmitted symbols are updated only when the estimates obtained in the current iteration provide an improvement over those from the previous iteration. This ensures that the algorithm consistently refines its results and avoids unnecessary updates that do not enhance the quality of the symbol detection.
**Algorithm 1:** ADL-MPA**Input:** Received signal $y_{\mathrm{DD}}$, equivalent channel matrix $H_{\mathrm{DD}}$
**Output:** Estimated transmitted symbols $\hat{x}\left[c\right]$
1: Initialize message $P_{c,d}^{0}=\frac{1}{Q}$ for c=1,…,NM, d∈J(c), and set iteration index i=1
2: **repeat**
3: **for** each observation node y[d] **do**
4:  Compute the mean $\mu_{d,c}^{\left(i\right)}$ and variance ${\left(\sigma_{d,c}^{\left(i\right)}\right)}^{2}$ of the Gaussian variable $\zeta_{d,c}^{\left(i\right)}$ using $P_{c,d}^{i-1}$
5:  Pass $\mu_{d,c}^{\left(i\right)}$ and ${\left(\sigma_{d,c}^{\left(i\right)}\right)}^{2}$ to variable nodes x[c], c∈I(d)
6: **end for**
7: **for** each variable node x[c] **do**
8:  Use (20), (21) and (23) to calculate the log-likelihood $\ln\xi^{\left(i\right)}$, the LSE normalized log-probability $\ln{\tilde{p}}_{c,d}^{\left(i\right)}$, and the adaptive damping factor $\Delta^{\left(i\right)}$
9:  Update the PMF $P_{c,d}^{\left(i\right)}$ use (22)
10:  Pass $P_{c,d}^{\left(i\right)}$ to observation nodes y[d], d∈J(c)
11: **end for**
12: Compute the convergence metric $\eta^{\left(i\right)}$
13: **if** $\eta^{\left(i\right)}>\eta^{\left(i-1\right)}$ **then**
14:  Update detected symbols using (26)
15: **end if**
16: i←i+1
17: **until** stopping criteria are satisfied
18: **return** $\hat{x}\left[c\right]$


The algorithm is designed to terminate when any of the following conditions is satisfied:

1. Convergence is achieved. Specifically, the convergence indicator $\eta^{\left(i\right)}=1$, signifying that the algorithm has fully converged to a stable solution where no further significant updates are necessary.
2. Significant degradation in performance. Specifically, the convergence indicator $\eta^{\left(i\right)}<\eta^{\left(j\right)}-\epsilon$, where $\eta^{\left(j\right)}$ is the maximum convergence indicator observed across all previous iterations j=1,…,i−1. Here, ϵ is a predefined threshold, set to 0.2. This condition ensures that the algorithm halts if the current iteration shows a substantial decline in performance compared to the best result achieved so far, preventing unnecessary computations and avoiding divergence.
3. Maximum iteration limit. Specifically, the algorithm reaches the predefined maximum number of iterations, which acts as a safeguard to ensure computational efficiency and prevent the algorithm from running indefinitely. This condition is particularly useful in scenarios where convergence is slow or cannot be achieved due to challenging system conditions.

The computational complexity of the proposed ADL-MP algorithm is significantly reduced compared to that of full MAP detection, which grows exponentially with the problem size. This reduction in complexity is achieved by leveraging the sparse structure of the channel and the iterative nature of message passing. The channel matrix $H_{\mathrm{DD}}$ is highly sparse, with only *P* non-zero entries per row and column. This sparsity reduces the number of computations required for each node from O(NM) to O(P), where P≪NM. For each observation node y[d], the mean $\mu_{d,c}^{\left(i\right)}$ and variance $\sigma_{d,c}^{\left(i\right)}$ of the interference term are computed by summing over *P* terms, leading to a complexity of O(P). For each variable node x[c], the log-domain message $\ln{\tilde{p}}_{c,d}^{\left(i\right)}$ is updated using LSE normalization, which involves summing over *Q* constellation points and *P* connections, resulting in a complexity of O(PQ). The adaptive damping factor $\Delta^{\left(i\right)}$ is calculated using the convergence indicator $\eta^{\left(i\right)}$. The computation of $\eta^{\left(i\right)}$ involves summing over all variable nodes and their connected edges, resulting in a complexity of O(NMP). Considering NM variable nodes and NM observation nodes, and assuming each node has *P* connections, the total complexity per iteration is O(NMPQ), where *Q* is the size of the symbol constellation and P≪NM due to channel sparsity. The number of iterations required for convergence depends on the channel conditions and signal-to-noise ratio (SNR). In practical scenarios, the algorithm typically converges within a small number of iterations. Thus, the overall complexity is approximately O(KNMPQ), where *K* is the number of iterations.

## 4. Simulation Results

In this section, the BER performance of the proposed ADL-MP detection algorithm is evaluated under the EVA channel model and the HST scenario. These scenarios were selected to represent realistic and challenging V2X wireless communication environments. The BER performance of the ADL-MP detection algorithm is compared to that of the conventional linear minimum mean square error (LMMSE) and maximal ratio combining (MRC) [25] detection algorithms. Then, we compare the bit error rate performance of FTN-OTFS and OFDM under the EVA model. The simulation parameters used in the simulations are summarized in Table 1, offering a comprehensive overview of the system configuration.

In this section, the BER performance of the proposed ADL-MP detection algorithm is evaluated under both the EVA channel model and the HST scenario, which were chosen to represent realistic and challenging V2X wireless communication environments. The BER performance of the ADL-MP algorithm is compared with that of conventional detection algorithms, specifically linear minimum mean square error (LMMSE) and maximal ratio combining (MRC) [25]. In addition, we compare the BER performance of FTN-OTFS and OFDM under the EVA model. The simulation parameters are summarized in Table 1, providing a comprehensive overview of the system configuration.

**Figure 3:**
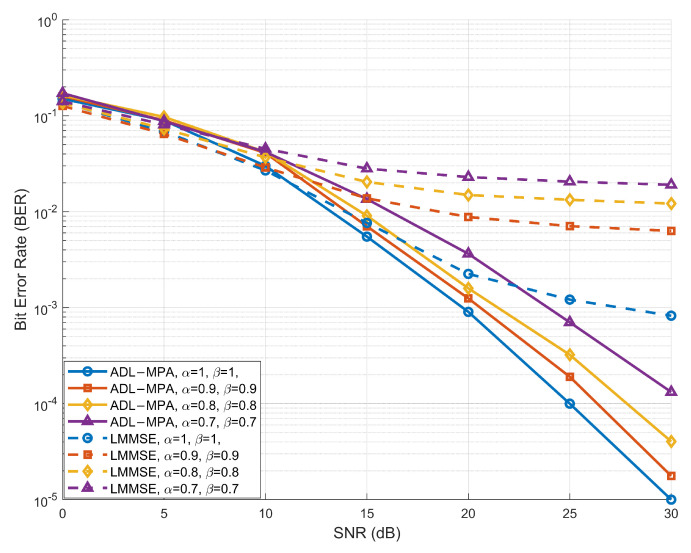
BER performance of the proposed scheme based on ADL-MPA versus LMMSE under different compression factors in the EVA model.

Figure 3 illustrates the BER performance of the proposed ADL-MP detection algorithm under the EVA channel model (In the EVA model, the delay taps are [0,30,150,310,370,710,1090,1730,2510] ns, with the Doppler shift for the *i*th path generated from a uniform distribution $U(0,\nu_{\max})$ [25], where $\nu_{\max}$ denotes the maximum Doppler shift) with a speed of 120 kmph. The results compare the BER performance of the ADL-MP algorithm and the conventional LMMSE detection algorithm across different compression factor pairs (α,β) over a range of SNR values. As depicted in Figure 3, the LMMSE and ADL-MP algorithms achieve comparable performance in the low-to-medium SNR range (0 to 15 dB). However, beyond 15 dB, the ADL-MP algorithm consistently outperforms LMMSE, delivering significantly lower values of BER. This improvement highlights the superior capability of ADL-MP to suppress noise and interference in highly dynamic multipath environments, such as those encountered in V2X communication systems. When the compression factors are set to (α,β)=(1,1), the system behaves equivalently to a conventional OTFS system. Under these conditions, the ADL-MP and LMMSE algorithms perform comparably at low and moderate SNR levels. However, as (α,β) decrease, the ADL-MP algorithm manages to maintain a BER below $10^{-3}$ across the high-SNR range, satisfying the requirement for reliability in V2X communication. In contrast, the BER performance of the LMMSE algorithm degrades significantly at higher compression rates, particularly in the high-SNR range, compromising system reliability.

**Figure 4:**
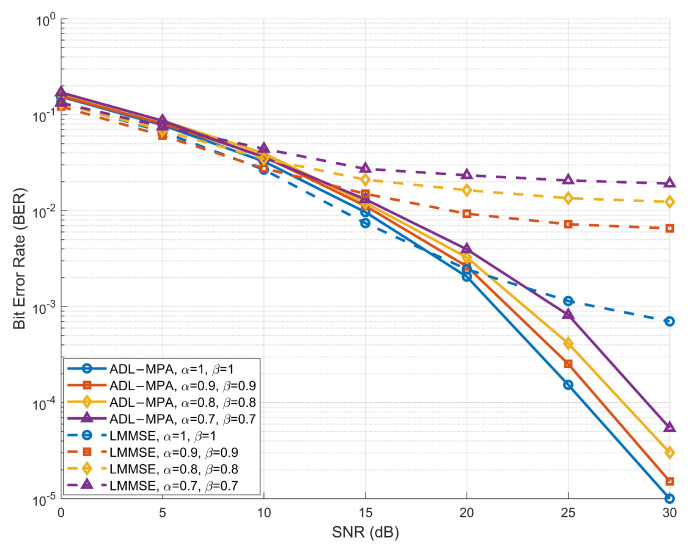
BER performance of the proposed scheme based on ADL-MPA versus LMMSE with different compression factors in the HST scenario.

Figure 4 illustrates the BER performance of the proposed ADL-MP detection algorithm under the HST scenario (The HST scenario is modeled as a single-path line-of-sight channel with zero delay, where the Doppler shift is set to the maximum value $\nu_{\max}$ determined by the train speed and carrier frequency [26]). The system is evaluated at a high speed of 500 km/h to represent the challenging conditions created by high mobility in vehicular communication systems. As shown in Figure 4, the BER performance of the ADL-MP algorithm is compared to that of the conventional LMMSE detection algorithm across different compression factor pairs (α,β) over a wide range of SNR values. Yielding results similar to those from the EVA model, the ADL-MP and LMMSE algorithms perform comparably in the low-to-medium-SNR range ([0, 15) dB). However, at higher SNR values ([15, 30] dB), the ADL-MP algorithm demonstrates a clear performance advantage, effectively suppressing noise and interference in the high-mobility LoS channel conditions of the HST scenario. For the case in which (α,β)=(1,1), the system corresponds to a traditional OTFS system, and the two detection algorithms exhibit similar performance at low and moderate SNR levels. As (α,β) decrease, the ADL-MP algorithm maintains a BER below $10^{-3}$ even at higher SNR values, meeting the stringent reliability requirements of high-speed V2X communication. Conversely, the BER performance of the LMMSE algorithm deteriorates significantly at higher compression rates, particularly at high SNR values, leading to reduced system reliability.

**Figure 5:**
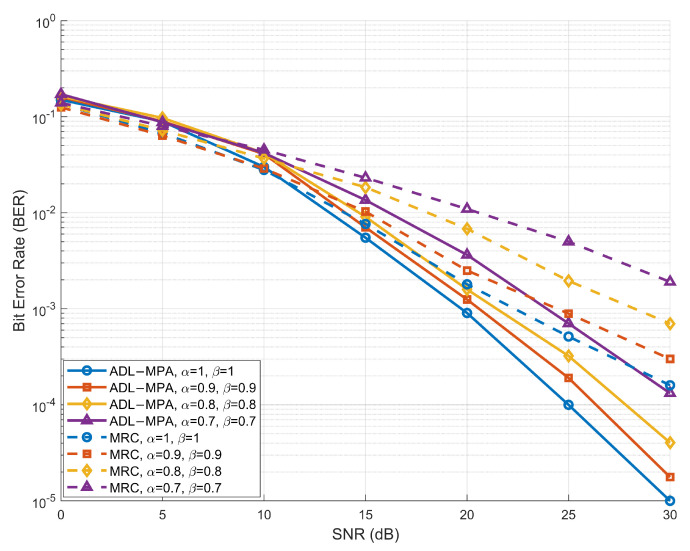
BER performance of the proposed scheme based on ADL-MPA versus MRC with different compression factors in the EVA model.

**Figure 6:**
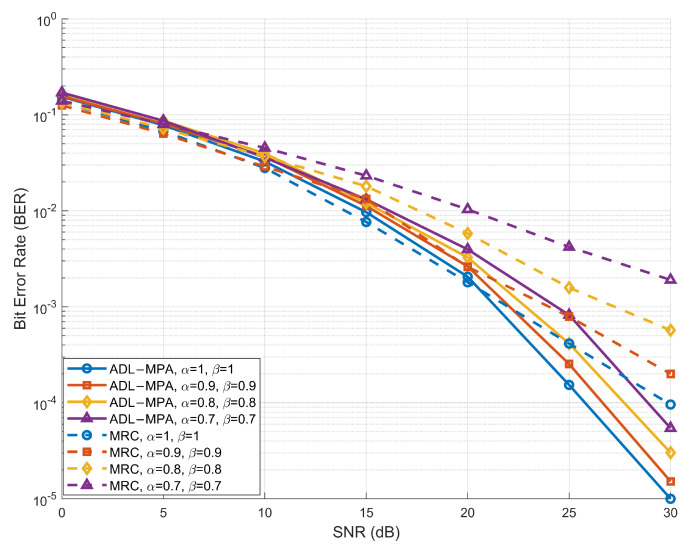
BER performance of the proposed scheme based on ADL-MPA versus MRC with different compression factors in the HST scenario.

Figure 5 and Figure 6 illustrate the BER performance of the proposed scheme based on the ADL-MPA algorithm versus the MRC algorithm [25] with various compression-factor pairs (α,β), in the EVA channel model and the HST scenario, respectively. In both cases, the proposed ADL-MPA algorithm consistently outperforms the MRC method across the entire SNR range for all considered values of the compression factors. Specifically, when α=β=0.9, the proposed scheme achieves an SNR gain of nearly 5 dB over the MRC algorithm at a BER of $10^{-3}$ in both channel environments. Moreover, the performance advantage becomes even more pronounced as the values of the compression factors decrease, demonstrating the robustness and effectiveness of the proposed algorithm under varying high-mobility conditions.

**Figure 7:**
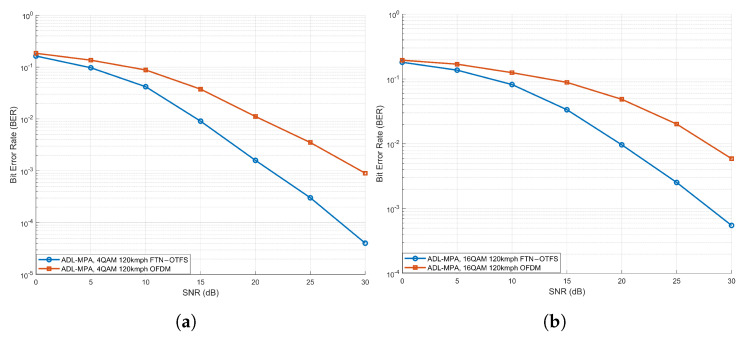
BER performance comparison between FTN-OTFS and OFDM under the EVA channel model. (**a**) 4QAM, (**b**) 16QAM.

Figure 7 presents a comparison of the BER performance between the proposed FTN-OTFS system and conventional OFDM (For fair comparison, the simulation parameters of OFDM were chosen to match those of FTN-OTFS. For example, both systems utilize M=64 and N=64 per frame. This comparison was carried out) under the EVA channel model at a vehicle speed of 120 kmph, utilizing the ADL-MP detection algorithm for both 4QAM and 16QAM modulations. As illustrated, with 4QAM modulation, the FTN-OTFS system achieves an SNR gain of more than 5 dB over OFDM at a BER of $10^{-3}$. Similarly, under 16QAM modulation, the proposed system also exhibits significant performance improvements compared to the OFDM system. These simulation results conclusively demonstrate the superior robustness of the proposed FTN-OTFS system in high-mobility V2X communication scenarios.

The simulation results demonstrate the robustness of the ADL-MP algorithm, especially in challenging compression scenarios and at high SNR levels. These findings emphasize the critical role of optimized detection in ensuring the reliability and efficiency of communication systems in dynamic vehicular environments.

## 5. Conclusions

In this paper, we propose a novel FTN-OTFS modulation scheme that integrates FTN signaling with SEFDM for high-mobility V2X communications. We derive analytically tractable input–output models in both the DD and TF domains, providing a solid foundation for efficient receiver design. To tackle the challenges of non-orthogonal transmission and mitigate the resulting interference, we propose an advanced ADL-MP detection algorithm. This algorithm was specifically designed to improve numerical stability, ensuring robust performance even in complex communication environments. Simulation results not only demonstrate the superiority of the proposed FTN-OTFS scheme over OFDM in high-mobility V2X communication, but also verify the effectiveness of the proposed ADL-MP algorithm in both the EVA channel model and HST scenarios. In conclusion, the proposed FTN-OTFS modulation scheme, combined with the ADL-MP detection algorithm, offers a powerful and reliable solution for future high-mobility V2X communication systems. Its superior performance and robustness make it a promising candidate to meet the stringent demands of next-generation vehicular networks.

## Figures and Tables

**Table 1:** Simulation Parameters.

Number of Subcarriers *M*	64
Number of Time Slots *N*	64
Mapping	4-QAM
Subcarrier Bandwidth	15 kHz
Channel	EVA and HST
Vehicular Speed	120 kmph and 500 kmph
Carrier Frequency	4 GHz
ADL-MP Iteration Count	15

## Data Availability

The original contributions presented in this study are included in the paper.

## Abbreviations

The following abbreviations are used in this manuscript:

V2X	vehicle-to-everything
OTFS	orthogonal time frequency space
FTN	faster-than-Nyquist signaling
SEFDM	spectrally efficient frequency division multiplexing
BER	bit error rate
ADL-MP	adaptive damping log-domain message passing
EVA	extended vehicular A model
HST	high speed train scenario
OFDM	orthogonal frequency division multiplexing
IDI	inter-Doppler interference
ICI	inter-carrier interference
ISI	inter-symbol interference
DD	delay-Doppler
EVD	eigenvalue decomposition
TF	time-frequency
FFT	fast Fourier transform
IFFT	inverse fast Fourier transform
ISFFT	inverse symplectic finite Fourier transform
DFT	discrete Fourier transform
IDFT	inverse discrete Fourier transform
IFrFT	inverse fractional Fourier transform
AWGN	additive white Gaussian noise
LSE	log-sum-exp
MAP	maximum a posteriori
